# A case–control study of selenoprotein genes polymorphisms and autoimmune thyroid diseases in a Chinese population

**DOI:** 10.1186/s12881-017-0415-6

**Published:** 2017-05-12

**Authors:** Ling Xiao, Jianghong Yuan, Qiuming Yao, Ni Yan, Ronghua Song, Wenjuan Jiang, Danfeng Li, Liangfeng Shi, Jin-an Zhang

**Affiliations:** 1 Department of Endocrinology, Jinshan Hospital of Fudan University, No. 1508 Longhang Road, Shanghai, 201508 People’s Republic of China; 2 Department of Nephrology, People’s Hospital of Tongchuan city, No. 12, Jiankang Street, Wangyi District, Tongchuan City, Shaanxi Province 727000 People’s Republic of China

**Keywords:** Selenoprotein P gene (SELENOP), Glutathione peroxidase 4 gene (GPX4), Selenoprotein S gene (SELENOS), Single-nucleotide polymorphisms (SNPs), Autoimmune thyroid disease (AITD)

## Abstract

**Background:**

Selenium is an essential trace and there is a high selenium concentration in the thyroid gland. Selenium deficiency may impair the thyroid function. The aim of this study was to investigate the association between three selenoprotein genes polymorphisms and autoimmune thyroid diseases.

**Methods:**

We genotyped six single-nucleotide polymorphisms (SNPs), rs6865453 in selenoprotein P gene (SELENOP), rs713041 rs2074451 rs3746165 in glutathione peroxidase 4 gene (GPX4) and rs28665122 and rs7178239 in selenoprotein S gene (SELENOS) by MassARRAY system using the chip-based matrix-assisted laser desorption ionization time-of-flight mass spectrometry technology in 1060 patients with autoimmune thyroid diseases and 938 healthy controls.

**Results:**

Major alleles in rs6865453 of SELENOP, rs713041, rs2074451, rs3746165 of GPX4 decreased while the major allele C in rs28665122 of SELENOS increased in AITD patients than in the control. The allele C and genotype CC in rs7178239 of SELENOS showed different trend in GD and HT patients when compared with the control. All the distribution difference showed nonsignificant. Analysis according to clinical features including ophthalmopathy, hypothyroidism and family history came out to be negative either.

**Conclusions:**

Our findings suggest non-association between three selenoprotein genes and AITD, conflicting to the positive result in another population. Different selenium nutrition status in different populations may contribute to conflicting results, the contribution of genetic variants in AITD mechanism may be another reason.

## Background

Selenium is an essential trace element and its distribution in nature varies widely. The soil of central and southern European countries is generally poor in selenium content, resulting in mild regional selenopenia, whereas in most North American regions it is abundant. Severe selenopenia was observed in large areas of central Asia. Selenium is present in the active site of selenoproteins, which are crucial to immune function, inflammation, redox processes and important for thyroid hormone synthesis and metabolism. There is a high selenium concentration in the thyroid gland, and selenium deficiency may impair the thyroid function. Reactive oxygen species (ROS), formed from thyroid hormone metabolism, may cause damage to different cellular structures and trigger tissue inflammation. Selenoproteins, which are powerful antioxidant enzymes, mitigate the effects of oxidative stress by elimination of ROS. In addition, selenoproteins play a vital role in the regulation of human immune system and Se deficiency is accompanied by dysregulation of both cell-mediated immunity and B cell function.

Autoimmune thyroid diseases (AITD), mainly including Graves’ disease (GD) and Hashimoto’s thyroiditis (HT), are the most frequent autoimmune disorders. GD is characterized clinically by hyperthyroidism, diffuse goiter and the presence of thyrotropin receptor antibodies (TRAb), while HT is characterized by apoptosis of thyrocytes leading to hypothyroidism and the presence of thyroid peroxidase antibodies (TPOAb) or antibodies against thyroglobulin (TGAb). Autoimmune thyroid disease (AITD) is a multifactorial disease in which autoimmunity against thyroid antigens develops against a particular genetic background facilitated by exposure to environmental factors. Environmental factors include smoking, vitamin D, iodine, selenium and so on. GD and HT also share some but not all susceptible genes.

The value of selenium supplementation in autoimmune thyroid disorders had been investigated and most studies confirmed the beneficial effect of selenium supplementation in Hashimoto’s and Graves’s diseases, though some studies showed conflicting results as well [1–11]. Recently, selenium was proved to be effective in mild inflammatory thyroid associated orbitopathy (TAO) too [12]. Different selenium nutrition state in different studies may partly explain different reactivity to the selenium supplementation, different genetics factors in different populations may be another reason. Few studies were done about the association of selenoprotein genetic variations in AITD.

The role for selenium supplementation in the treatment of autoimmune thyroid diseases is still unresolved. Clinic studies provide evidence for a preventive effect of selenium on autoimmune thyroid disease, which may be mediated by the antioxidative and anti-inflammatory properties of selenoproteins, we therefore investigated whether genetic variants in selenoproteins are associated with GD and HT. Among 25 selenoproteins known to date, various functions are characterized for each of them. These include Se transport (selenoprotein P), antioxidant/redox properties (glutathione peroxidases (GPxs), thioredoxin reductases and selenoprotein P) and anti-inflammatory properties (selenoprotein S and GPx4). We chosed to study SNPs rs6865453 in selenoprotein P gene (SELENOP), rs713041 rs2074451 rs3746165 in glutathione peroxidase 4 gene (GPX4), rs28665122 and rs7178239 in selenoprotein S gene (SELENOS) in a Han Chinese population.

## Methods

### Subjects

Our study population (*n* = 1998) consisted of 1060 Chinese AITD patients including 701 GD patients, 359 HT patients and 938 healthy unrelated controls. All AITD patients in the present case–control study were recruited from the Department of Endocrinology, Jinshan Hospital Affiliated to Fudan University. The diagnostic criteria for GD were mainly determined by clinical manifestation, laboratory biochemical proof of hyperthyroidism, the presence of diffuse goiter and the positive circulating TSH receptor antibody (TRAb). HT was defined on the basis of enlarged thyroid and the high level of either TPOAb or TgAb, with or without clinical and biochemical hypothyroidism. All healthy controls were unrelated individuals with neither thyroid diseases nor other autoimmune diseases, recruited from the Medical Examination Center of the same hospital. All the subjects, including AITD patients and controls, were Han Chinese and signed the informed consent. Both patients and controls have similar diet habits and individuals with distinct diet style such as vegetarians were excluded. The research project was approved by the Ethics Committee of Jinshan Hospital Affiliated to Fudan University.

### Genotyping

Peripheral venous blood of 2 ml from the subjects was collected in an EDTA tube. The genomic DNA was extracted by salting-out method, using RelaxGene Blood DNA System (TIANGEN BIOTECH, Beijing, China), according to the manufacturers’ protocol. Genotyping of SNPs (rs6865453 in SELENOP, rs713041, rs2074451 and rs3746165 in GPX4, rs28665122 and rs7178239 in SELENOS) was performed by MassARRAY system (Sequenom, San Diego, CA, USA) using the chip-based matrix-assisted laser desorption ionization time-of-flight mass spectrometry technology. This is a well-selected platform by many researchers during studies including SNP genotyping. It has high specificity and sensitivity. Primers were obtained from Sangon Biotech (Shanghai, China). Briefly, multiplex reaction was designed using Assay Designer software version 3.0 (Sequenom) and was processed following standard protocols for iPLEX chemistry. The reaction products were then cleaned and dispensed onto a Spectro-CHIP bioarray. The chip was scanned using MassARRAY workstation version 3.3, and the resulting spectra were analyzed using the Sequenom TYPER software.

### Statistical analysis

We evaluated the Hardy-Weinberg equilibrium of SNPs within our population using the HWE program [13]. Allele and genotype frequencies between cases and controls were computed by chi-square test or Fisher’s exact test. Differences between groups were determined by the odds ratio (OR) and 95% confidence interval (95%CI). These statistical analyses were performed using the software GraphPad Prism 6. Rectified analysis between cases and controls for ages were conducted by Binary Logistic regression analysis using the software SPSS22.0. A p value less than 0.05 was considered significant.

## Results

### Clinical data analysis

As shown in Table 1, our study investigated 1060 AITD patients, who comprised 701 GD (214 men and 487 women) and 359 HT patients (44 men and 315 women). In GD patients, the average onset age was 33.86 ± 14.36, 145 individuals had family history and 154 had ophthalmopathy. In HT patients, the average onset age was 32.63 ± 13.44, 72 individuals had family history and 6 had ophthalmopathy. In addition, 184 patients suffered from hypothyroidism while other 175 were of euthyroidism in the HT group.

**Table 1:** Clinical data of AITD patients and controls

	GD	HT	Control
Number	701	359	938
Gender			
Female	487	315	629
Male	214	44	309
Age	36.88 ± 14.56	35.01 ± 13.81	38.75 ± 9.14
Onset of age	33.86 ± 14.36	32.63 ± 13.44	
Thyroid size			
Normal	25	13	938
I	133	63	
II	429	247	
III	114	36	
Family history			
(+)	145	72	
(−)	501	273	938
Ophthalmopathy			
(+)	154	6	
(−)	547	353	
Hypothyroidism			
(+)		184	
(−)		175	

### Allele and genotype results

The distribution of genotype frequencies for each SNP (rs6865453/rs713041/rs2074451/rs3746165/rs28665122/rs7178239) was in Hardy–Weinberg equilibrium in both the patients and the controls (Table 2). The allele distributions of the 6 SNPs of three genes, their genotype and case–control association analysis were shown in (Table 3). The major allele A and AA genotype in SELENOP rs6865453, the major allele C and CC genotype in rs713041, the major allele G and GG genotype in rs2074451, and the major allele C and CC genotype in rs3746165 of GPX4 gene decreased in both GD and HT patients than those in the control. The major allele C and genotype CC in SELENOS rs7178239 showed different trend in GD and HT patients when compared to the control. While the major allele C and CC genotype in rs28665122 of SELENOS increased in both GD and HT patients than the control, different to the result for another study, in which these allele and genotype decreased significantly in their HT patients [14]. Our association analysis showed negative and the distribution difference showed nonsignificant.

**Table 2:** Hardy-Weinberg P value of the six SNPs

SNP ID	Control	GD	HT
rs6865453	0.2189	0.4966	0.0933
Rs713041	0.5782	0.5330	0.3827
Rs2074451	0.8859	0.6388	0.1908
Rs3746165	0.6388	0.8765	0.0829
Rs28665122	0.0576	0.9438	0.0546
Rs7178239	0.7108	0.9519	0.7774

**Table 3:** Allele and genotype frequencies in AITD patients and controls

GENE	SNP	Alleles	Con (%)	GD (%)	P	Adjusted OR (95%CI)	HT (%)	P	Adjusted OR (95%CI)
Sepp1	rs6865453	A	1338 (74.5)	983 (72.8)	0.307	0.918 (0.779–1.081)	514 (74.1)	0.813	0.975 (0.791–1.201)
		C	458 (25.5)	367 (27.2)			180 (25.9)		
		AA	491 (54.7)	354 (52.4)	0.403	0.918 (0.751–1.122)	184 (53.03)	0.605	0.936 (0.727–1.203)
		AC + CC	407 (45.3)	321 (47.6)			163 (47.0)		
GPX4	rs713041	C	1031 (57.4)	751 (55.6)	0.329	0.932 (0.809–1.073)	390 (56.2)	0.739	0.970 (0.810–1.160)
		T	765 (42.6)	599 (44.37)			304 (43.8)		
		CC	300 (33.4)	213 (31.6)	0.418	0.915 (0.739–1.133)	105 (30.3)	0.370	0.883 (0.674–1.158)
		CT + TT	598 (66.6)	462 (68.5)			242 (69.7)		
	rs2074451	G	1004 (56.0)	738 (55.2)	0.698	0.972 (0.843–1.120)	375 (54.66)	0.713	0.966 (0.806–1.158)
		T	790 (44.0)	598 (44.8)			311 (45.3)		
		GG	292 (31.4)	207 (31.0)	0.833	0.977 (0.786–1.213)	96 (27.99)	0.343	0.874 (0.662–1.154)
		GT + TT	615 (68.6)	451 (69.0)			247 (72.0)		
	rs3746165	C	1019 (56.7)	738 (54. 7)	0.259	0.921 (0.798)–1.062)	384 (55.33)	0.625	0.955 (0.796–1.146)
		T	777 (43.3)	612 (45.3)			310 (44.7)		
		CC	287 (32.0)	203 (30.1)	0.416	0.914 (0.736–1.135)	98 (28.24)	0.272	0.857 (0.650–1.129)
		CT + TT	611 (68.0)	472 (70.0)			249 (71.8)		
SEPS1	rs28665122	C	1688 (93.5)	1267 (94.4)	0.297	1.168 (0.872–1.566)	668 (95.16)	0.148	1.325 (0.904–1.943)
		T	118 (6.5)	75 (5.6)			34 (4.8)		
		CC	793 (87.8)	598 (89.1)	0.432	1.134 (0.828–1.554)	320 (91.2)	0.104	1.420 (0.930–21.67)
		CT + TT	110 (12.2)	73 (10.9)			31 (8.8)		
	rs7178239	G	802 (45.6)	620 (46.9)	0.496	1.051 (0.910–1.214)	291 (42.4)	0.167	0.881 (0.735–1.054)
		C	958 (54.4)	702 (53.1)			395 (57.6)		
		GG	180 (20.5)	145 (21.9)	0.514	1.085 (0.848–1.389)	63 (18.4)	0.370	0.863 (0.625–1.190)
		CG + CC	700 (79.6)	516 (78.1)			280 (81.6)		

### Genotype, allele distribution and clinical phenotype association

Etiology for Graves’ ophthalmopathy remains unclear, and genetic factors contribute to it too. Here we compared the genotype, allele distribution of Graves’ ophthalmopathy patients or non-ophthalmopathy ones to the control and also did the association analysis between ophthalmopathy and non-ophthalmopathy patients, but no association was found (Table 4). When we did similar analysis between HT patients with hypothyroidism and the euthyroidism HT patients, no significant difference was found either (Table 5). We did the association analysis according to the patients’ family history too. The statistic results were negative in GD patients grouped on the basis of their family history (Table 6). When HT patients were divided into family history-positive and negative groups, the patients number was too small to do effective statistical analysis. So this part of analysis was ignored.

**Table 4:** Allele and genotype frequencies in GD patients with or without ophthalmopathy and controls

GENE	SNP	Alleles	Con (%)	GO (%)	P	non-GO (%)	P	P (GO/non-GO)
Sepp1	rs6865453	A	1338 (74.5)	173 (72.08)	0.380	710 (73.20)	0.462	0.7456
		C	458 (25.5)	67 (27.92)		260 (26.80)		
		AA	491 (54.68)	63 (52.5)		257 (52.99)		
		AC	356 (39.64)	47 (39.17)		196 (40.41)		
		CC	51 (5.68)	10 (8.33)		32 (6.60)		
GPX4	rs713041	C	1031 (57.41)	136 (56.67)	0.814	540 (55.67)	0.410	0.8277
		T	765 (42.59)	104 (43.33)		430 (44.33)		
		CC	300 (33.41)	35 (29.17)		156 (32.16)		
		CT	431 (48)	66 (55)		228 (47.01)		
		TT	167 (18.6)	19 (15.83)		101 (20.83)		
	rs2074451	G	1004 (55.96)	131 (55.51)	0.867	531 (55.31)	0.795	1
		T	790 (44.04)	105 (44.49)		429 (44.69)		
		GG	292 (31.44)	33 (27.97)		152 (31.67)		
		GT	440 (49.05)	65 (55.08)		227 (47.29)		
		TT	175 (19.51)	20 (16.95)		101 (21.04)		
	rs3746165	C	1019 (56.74)	128 (53.33)	0.290	537 (55.36)	0.541	0.6121
		T	777 (43.26)	112 (46.67)		433 (44.64)		
		CC	287 (31.96)	31 (25.83)		152 (31.34)		
		CT	445 (49.55)	66 (55)		233 (48.04)		
		TT	166 (18.49)	23 (19.17)		100 (20.62)		
SEPS1	rs28665122	C	1688 (93.47)	225 (93.75)	0.887	909 (94.69)	0.228	0.5304
		T	118 (6.53)	15 (6.25)		51 (5.31)		
		CC	793 (87.82)	105 (87.5)		430 (89.58)		
		CT	102 (11.3)	15 (1.25)		49 (10.21)		
		TT	8 (0.9)	0 (0)		1 (0.21)		
	rs7178239	G	802 (45.57)	117 (50.87)	0.121	443 (46.53)	0.711	0.2401
		C	958 (54.43)	113 (49.13)		509 (53.47)		
		GG	180 (20.45)	30 (26.09)		102 (21.43)		
		CG	442 (50.23)	57 (49.57)		239 (50.21)		
		CC	258 (29.32)	28 (24.34)		135 (28.36)

**Table 5:** Allele and genotype frequencies in HT patients with hypothyroidism or euthyroidism and controls

GENE	SNP	Alleles	Con (%)	HT-hypo (%)	P	HT-eu (%)	P	P (Eu/hypo)
SEPS1	rs28665122	C	1688 (93.47)	344 (95.56)	0.183	204 (93.58)	0.914	0.335
		T	118 (6.53)	16 (4.44)		14 (6.42)		
		CC	793 (87.82)	165 (91.67)		97 (88.99)		
		CT	102 (11.3)	14 (7.78)		10 (9.17)		
		TT	8 (0.9)	1 (0.55)		2 (1.84)		
	rs7178239	G	802 (45.57)	148 (42.05)	0.204	90 (42.86)	0.414	0.860
		C	958 (54.43)	204 (57.95)		120 (57.14)		
		GG	180 (20.45)	32 (18.18)		22 (20.95)		
		CG	442 (50.23)	84 (47.73)		46 (43.80)		
		CC	258 (29.32)	60 (34.09)		37 (35.25)		
Sepp1	rs6865453	A	1338 (74.5)	271 (75.70)	0.585	158 (73.83)	0.853	0.619
		C	458 (25.5)	87 (24.30)		56 (26.17)		
		AA	491 (54.68)	99 (55.31)		54 (50.47)		
		AC	356 (39.64)	73 (40.78)		50 (46.73)		
		CC	51 (5.68)	7 (3.91)		3 (2.80)		
GPX4	rs713041	C	1031 (57.41)	208 (58.10)	0.694	117 (54.67)	0.500	0.433
		T	765 (42.59)	150 (41.90)		97 (45.33)		
		CC	300 (33.41)	59 (32.96)		29 (27.10)		
		CT	431 (48)	90 (50.28)		59 (55.14)		
		TT	167 (18.6)	30 (16.76)		19 (17.76)		
	rs2074451	G	1004 (55.96)	200 (56.50)	0.725	110 (52.38)	0.401	0.381
		T	790 (44.04)	154 (43.50)		100 (47.62)		
		GG	292 (31.44)	53 (29.94)		25 (23.81)		
		GT	440 (49.05)	94 (53.11)		60 (57.14)		
		TT	175 (19.51)	30 (16.95)		20 (19.05)		
	rs3746165	C	1019 (56.74)	204 (56.98)	0.816	114 (53.27)	0.399	0.434
		T	777 (43.26)	154 (43.02)		100 (46.73)		
		CC	287 (31.96)	55 (30.73)		26 (24.30)		
		CT	445 (49.55)	94 (52.51)		62 (57.94)		
		TT	166 (18.49)	30 (16.76)		19 (17.76)

**Table 6:** Allele and genotype frequencies in GD patients with or without family history and controls

GENE	SNP	Alleles	Con (%)	GD-FH (%)	P	GD non-FH (%)	P	P (FH/non-FH)
Sepp1	rs6865453	A	1338 (74.5)	206 (75.18)	0.675	694 (71.55)	0.089	0.2517
		C	458 (25.5)	68 (24.82)		276 (28.45)		
		AA	491 (54.68)	77 (56.20)		245 (50.52)		
		AC	356 (39.64)	52 (37.96)		204 (42.06)		
		CC	51 (5.68)	8 (5.84)		36 (7.42)		
GPX4	rs713041	C	1031 (57.41)	151 (55.11)	0.511	540 (55.67)	0.393	0.8906
		T	765 (42.59)	123 (44.89)		430 (44.33)		
		CC	300 (33.41)	39 (28.47)		155 (31.96)		
		CT	431 (48)	73 (53.28)		230 (47.42)		
		TT	167 (18.6)	25 (18.25)		100 (20.62)		
	rs2074451	G	1004 (55.96)	150 (55.56)	0.980	529 (55.10)	0.680	0.9448
		T	790 (44.04)	120 (44.44)		431 (44.90)		
		GG	292 (31.44)	39 (28.89)		149 (31.04)		
		GT	440 (49.05)	72 (53.33)		231 (48.13)		
		TT	175 (19.51)	24 (17.78)		100 (20.83)		
	rs3746165	C	1019 (56.74)	146 (53.28)	0.353	535 (55.15)	0.438	0.5833
		T	777 (43.26)	128 (46.72)		435 (44.85)		
		CC	287 (31.96)	36 (26.28)		150 (30.93)		
		CT	445 (49.55)	74 (50.01)		235 (48.45)		
		TT	166 (18.49)	27 (19.71)		100 (20.62)		
SEPS1	rs28665122	C	1688 (93.47)	265 (95.32)	0.244	907 (94.87)	0.161	0.8764
		T	118 (6.53)	13 (4.68)		49 (5.13)		
		CC	793 (87.82)	126 (90.65)		431 (90.17)		
		CT	102 (11.3)	13 (9.35)		45 (9.41)		
		TT	8 (0.9)	0 (0)		2 (0.42)		
	rs7178239	G	802 (45.57)	119 (44.40)	0.609	443 (46.83)	0.542	0.4886
		C	958 (54.43)	149 (55.60)		503 (53.17)		
		GG	180 (20.45)	27 (20.15)		101 (21.35)		
		CG	442 (50.23)	65 (48.51)		241 (50.95)		
		CC	258 (29.32)	42 (31.34)		131 (27.70)		

## Discussion

Selenium is incorporated into selenoproteins implicated in antioxidative defence, thyroid hormone metabolism and immunoregulation. Selenoproteins are important for the thyroid function and the thyroid health. Selenium metabolism is controlled by hepatocytes synthesizing and secreting the Se transporter selenoprotein P (SePP). SePP, encoded by SELENOP gene, is a liver-derived plasma protein and the major selenium containing protein in serum. Serum levels of SePP in humans are considered a biomarker of selenium status in the entire body. Its promoter activity has been shown to be inhibited by cytokines including interleukin 1β, tumor necrosis factor α, interferon γ and transforming growth factor β1 [15, 16], suggesting a role of SELENOP in the process of inflammation. SePP is able to bind to the endothelium and be recruited to the site of the inflammation. It also has the ability to bind epithelial cells and displays phospholipid hydroperoxide thiol peroxidase activity [17]. Overexpression of SELENOP suppressed hydrogenperoxide-induced activation of JNK and p38 whereas the gene’s knocked-down led to increased production of lipid hydroperoxide [18]. Studies showed that genetic variants in SELENOP were associated with the risk of breast cancer, prostate cancer, advanced colorectal adenoma and colorectal cancer as well as first phase insulin response and the occurrence of abdominal aortic aneurysm [19–24]. No studies about their roles in AITD were reported. We detected the association of rs6865453 in SELENOP with AITD, but it was negative.

Phospholipid hydroperoxide glutathione peroxidase (PHGPx, GPx4) is an intracellular selenoprotein, a major antioxidant enzyme, which plays unique roles in the protection of cells against oxidative stress by catalysing reduction of lipid hydroperoxides. GPX4 may also play a role in regulation of leukotriene biosynthesis and cytokine signaling pathways [25, 26]. GPx4 rs713041 polymorphisms could modulate the synthesis and expression of GPx4 by altering the selenocysteine insertion [27, 28]. GPx4 rs713041 were found to be associated with breast cancer mortality, the risk of colorectal cancer and cerebral stroke [23, 29, 30]. GPx4 rs2074451 variant was also found to be associated with breast cancer survival [24]. And rs3746165 variants in GPx4 may be associated with risk of lethal prostate cancer [31]. No relevance of these SNPs to AITD was reported. We detected these three GPx4 SNPs in this case–control study, but no association with AITD was found.

Selenoprotein S (SELENOS, gene aliases: SELS, VIMP, TANIS) has been classified as a new endoplasmic reticulum (ER) membrane protein that moves misfolded proteins from the ER to the cytosol and prevents stress responses that activate the inflammatory cascade [32]. It is a membrane-bound selenoprotein and located in ER and other plasma membrane. It protects cells from oxidative damage and it impacts the immune and inflammatory signal pathways. It is expected that genetic polymorphisms affecting SELENOS gene transcription and subsequent SELENOS expression levels might contribute to the development and progression of inflammatory disorders. A previous study discovered that a functional single nucleotide polymorphism (SNP) in the SELENOS promoter region (G-105A, rs28665122) impairs SELENOS expression and amplifies the production of inflammatory cytokines such as tumor necrosis factor-a (TNF-a), interleukin-6 (IL-6) and interleukin-1b (IL-1b) [33]. No significant differences were observed in the genotypic distribution of the SELENOS polymorphisms analyzed in Spanish cohorts between patients suffering from autoimmune diseases with a clear inflammatory component, like T1D, RA or IBD and controls [34]. While the SELENOS rs28665122 polymorphism had been found to be associated with the risk of spontaneous preterm birth in a Chinese population [35], the existence of association between this genetic variation and HT risk was also found in a Portuguese population with a higher allele A proportion in the patient group [14]. Here we detected the link between rs28665122 and rs7178239 polymorphisms in SELENOS gene and AITD in a Chinese population. Unfortunately, the association between AITD risk and these two SELENOS SNPs in our study was negative. Opposing to the result from the Portuguese population, the allele C and CC genotype in SELENOS rs28665122 increased in HT patients in our study. Inconsistent result suggests that different genetic variants may be involved in the etiology of AITD in different populations. Different pathogenic mechanisms may underlie autoimmune thyroid disease in different populations. This may partly explain the conflicting effects of selenium administration on AITD antibodies in studies from different populations. Different selenium nutrition status of the population from different geographical location may be another factor contributing to this inconsistent result. Clearly, further in-depth studies and evaluation are required concerning the mechanism of selenium action and the etiology of AITD.

## Conclusions

Our findings suggested non-association between six SNPs of three selenprotein genes and AITD, non-association between SNPs and clinical genotypes. Our results were conflicted to the result from the Portuguese population study. In that study, the higher allele A in SELENOS rs28665122 had been found to be associated with the HT risk [14]. Different selenium nutrition state in different populations may contribute to conflicting results, the contribution of genetic variants in AITD mechanism may be another reason.

## Abbreviations

AITD: Autoimmune thyroid disease
CI: Confidence interval
ER: Endoplasmic reticulum
GD: Graves’ disease
GPX4: Glutathione peroxidase 4 gene
GPx4: Phospholipid hydroperoxide glutathione peroxidase
HT: Hashimoto’s thyroiditism
IBD: Inflammatory bowel disease
IL-1b: Interleukin-1b
IL-6: Interleukin-6
OR: Odds ratio
RA: Rheumatoid arthritis
ROS: Reactive oxygen species
SELENOP: Selenoprotein P gene
SELENOS: Selenoprotein S gene
SePP: Se transporter selenoprotein P
SNPs: Single-nucleotide polymorphisms
T1D: Type 1 diabetes
TAO: Thyroid associated orbitopathy
TGAb: Antibodies against thyroglobulin
TNF-a: Tumor necrosis factor-a
TPOAb: Thyroid peroxidase antibodies
TRAb: Thyrotropin receptor antibodies
